# Pro-Inflammatory Th1 and Th17 Cells Are Suppressed During Human Experimental Endotoxemia Whereas Anti-Inflammatory IL-10 Producing T-Cells Are Unaffected

**DOI:** 10.3389/fimmu.2018.01133

**Published:** 2018-05-18

**Authors:** Alexandra Brinkhoff, Annette Sieberichs, Harald Engler, Sebastian Dolff, Sven Benson, Johannes Korth, Manfred Schedlowski, Andreas Kribben, Oliver Witzke, Benjamin Wilde

**Affiliations:** ^1^Department of Nephrology, University Hospital Essen, University of Duisburg-Essen, Essen, Germany; ^2^Institute of Medical Psychology and Behavioral Immunobiology, University Hospital Essen, University of Duisburg-Essen, Essen, Germany; ^3^Department of Infectious Diseases, University Hospital Essen, University of Duisburg-Essen, Essen, Germany

**Keywords:** systemic inflammation, T-cells, IL-17A, IFNγ, Treg, IL-10, endotoxemia

## Abstract

**Objective:**

Sepsis is one of the leading causes of the deaths in hospitals. During sepsis, patients are exposed to endotoxemia, which may contribute to the dysregulation of the immune system frequently observed in sepsis. This dysregulation leads to impaired pro-inflammatory responses and may increase the risk for secondary infections in sepsis. The experimental human endotoxemia model is widely used as a model system to study the acute effects of endotoxemia. Under physiological circumstances, the immune system is tightly regulated. Effector T-cells exert pro-inflammatory function and are restrained by regulatory T-cells (Tregs), which modulate pro-inflammatory effector responses. Endotoxemia may induce inadequate Treg activity or render effector T-cells dysfunctional. It was the aim of the study to investigate effector T-cell and Treg responses in an experimental human endotoxemia model.

**Methods:**

In a cross-over designed placebo-controlled study, 20 healthy male volunteers received an intravenous injection of either lipopolysaccharide (LPS) (0.8 ng/kg body weight) or a placebo (saline 0.9%). CD3^+^ T-cells, CD4^+^ T-cells, CD8^+^ T-cells, and intracellular cytokine profiles were measured with flow cytometry at baseline and at repeated points after LPS/placebo injection. Complete blood cell counts were obtained with an automated hematology analyzer and cytokines were quantified by ELISA.

**Results:**

Circulating neutrophils were significantly increased 2 h after LPS injection (*p* < 0.001) while absolute number of CD3^+^ T-cells, CD4^+^ T-cells, and CD8^+^ T-cells decreased (*p* < 0.001). Effector T-helper-cells (THs) showed a significant—but transient—decrease of pro-inflammatory IFNγ, interleukin (IL)-2, TNFα, and IL-17A production after LPS injection (*p* < 0.001). In contrast, the frequency of Treg and the capacity to produce IL-10 were unchanged (*p* = 0.21).

**Conclusion:**

Effector THs fail to produce pro-inflammatory Th1-/Th17-associated cytokines after LPS challenge. In contrast, IL-10 production by Treg is not affected. Thus, endotoxemia-induced suppression of pro-inflammatory THs might be considered as a contributing factor to immunoparalysis in sepsis.

## Introduction

Sepsis is one of the leading causes of deaths in hospitals. Therapeutic options for sepsis patients are limited and mortality rates remain high (1–3). This life-threatening syndrome develops as a result of a dysregulated immune response to a pathogen (4). In this case, the clearance of the pathogen is inefficient and there is continuous activation of specific pro-inflammatory pathways (5). At the same time, the effector response of the immune system is disturbed; and the innate as well as the adaptive immune system are hypo-responsive (4, 5). In addition, a post-mortem study by Boomer et al. revealed that patients with fatal clinical course of sepsis showed signs of severe immunological dysfunction (6). Splenocytes had a reduced capacity to produce pro-inflammatory cytokines upon stimulation, and splenic T-cells were diminished in numbers (6). This so-called “immunoparalysis” is a severe immunosuppressive state that makes the host susceptible for secondary infections (4, 5, 7). Experimental human endotoxemia, in which lipopolysaccharide (LPS) is administered to healthy volunteers, has been established as a model to study the diverse effects of endotoxemia (8–12). In this model, features of dysfunctional immunity can be observed. Leukocytes from healthy volunteers with LPS exposure show reduced responsiveness to *ex vivo* stimulation with LPS and other toll-like-receptor agonists (7–11, 13). Therefore, the human experimental endotoxemia model was also used in a double-blind placebo-controlled pilot study to investigate agents, which may reverse immunoparalysis (7). Recent studies emphasize the growing importance of effector T-cells and regulatory T-cells (Tregs) in sepsis (14). Effector T-cell subsets have pro-inflammatory function, are classified according to signature cytokines, and have pivotal role in defense against pathogens—Th1 cells produce interferon gamma (IFNγ) and interleukin (IL)-2 to support cell-mediated immunity; Th17 cells produce IL-17 (Th17) and have a crucial role in the inflammatory response against parasites, extracellular, and fungal pathogens (15, 16). Treg subsets with anti-inflammatory capacity limit pro-inflammatory T-cell responses (17). Tregs balance T-cell homeostasis, activation, and function *via* numerous different mechanisms including secretion of IL-10 (17, 18). IL-10 has been suggested as an important regulator in sepsis (4, 19).

It has not been well studied which type of T-cell subsets are affected by endotoxemia, and the kinetics of the T-cell dysfunction are not exactly known. The aim of this study was to characterize T-cell responses during endotoxemia. Therefore, T-cell function of pro-inflammatory effector T-cells and anti-inflammatory Tregs was analyzed in a human endotoxemia model.

## Materials and Methods

### Participants

The study is a single-center, placebo-controlled, randomized, and single-blinded trial in a cross-over study design. Healthy men aged 18–40 years were recruited by public advertisement. The extensive screening and safety procedure consisted of personal interview, conducted by a physician, a physical examination including an assessment of blood and clinical chemistry parameters (complete blood cell count, C-reactive protein, coagulation factors, lactate-dehydrogenase, myoglobin, creatinkinase, liver enzymes, renal, and hormonal parameters). Laboratory screening was conducted before each study day (LPS vs. placebo) and up to 1 week after completing the study. Participants were excluded with reported or current medical and psychological conditions, body mass index (BMI) <19.0 or ≥ 27.0 kg/m^2^, current medication, smoking, regular and/or high alcohol consumption, severe allergies, or depression scores exceeding published cut-offs of the Beck Depression Inventory (BDI, 14). Additional excluding factors were extensive sport exercises 24 h before and after the study days and vaccinations within the last 2 months. One participant did not complete the +72 h time point within the LPS condition due to a case of family related acute gastroenteritis. The study protocol was approved by the local ethics committee of the University Hospital Essen, Germany (permission sign: 15-6533-BO). All volunteers provided written informed consent in accordance with the Declaration of Helsinki and received financial reimbursement for their participation in the study.

### Study Protocol

The study comprised a placebo and a LPS condition, i.e., participants received either LPS or placebo on two otherwise identical study days. The participants either started with the placebo condition followed by the LPS condition or with the LPS condition followed by the placebo condition, with a minimum of 7 days between study conditions. The order of study days was randomized and counterbalanced (www.randomizer.org was used for randomization). The study took place in a medically equipped room under supervision of an internal physician. After the arrival of the participants, an intravenous catheter was inserted in a cubital vein for repeated blood drawing and endotoxin injection. After a rest of 30 min, vital signs including heart and breathing rate, pulse oximetry (Kernmed Oled, Ettlingen, Germany), and blood pressure (Dinamap Compact T, Critikon, Norderstedt, Germany) were measured. One hour after arrival, subjects received an intravenous injection of 0.8 ng LPS/kg of body weight (*Eschericha coli* LPS 200 ng/ml, LOT HOK354, USP The United States Pharmacopeial Convention, Inc., Rockville, MD, USA as previously described) under continuous vital sign monitoring (20). LPS had been consigned to the German federal Agency for Sera and Vaccination (Paul-Ehrlich Institute, Langen, Germany) for a microbial safety testing and was stored in endotoxin-free borosilicate tubes (Pyroquant Diagnostik, Mörfelden-Walldorf, Germany) at −20°C until use. At study days, blood samples for complete blood counts and cytokine analysis were collected in EDTA-coated tubes at defined time points: before (baseline), as well as +1, +2, +3, +4, +6, +24, +48, and +72 h after the injection of endotoxin (LPS) or placebo, respectively. EDTA plasma was separated by centrifugation and stored at −80°C. Venous blood was collected in heparinized tubes at the following time points: baseline, +3, +24, +48, and +72 h. Following each blood sampling, body temperature, blood pressure, heart rate, and breathing rate were assessed.

### White Blood Cell Count

Complete blood counts containing white blood cell (WBC), neutrophils, mono-/lymphocytes, and platelets were obtained *via* automated hematology analyzer (KX-21N, Sysmex Deutschland GmbH, Norderstedt, Germany) using EDTA-anticoagulated peripheral blood samples.

### PBMC Isolation

PBMC isolation was facilitated by Ficoll density gradient centrifugation. The gradient was a commercially available Ficoll separation medium (Lymphoprep, STEMCELL Technologies, Köln, Germany). PBMCs were resuspended in RPMI 1640 medium with Glutamax (Gibco Life Technologies, Darmstadt, Germany) supplemented with 10% heat-inactivated fetal calf serum (Greiner Bio-One, Frickenhausen, Germany), 2% of penicillin and streptomycin, as well as with non-essential amino acids (MEM NEA) and sodium pyruvate (all from Gibco Life Technologies).

### Antibodies and Flow Cytometry

The fluorescently conjugated antibodies were supplied by BD Biosciences (BD Biosciences, Erembodegen, Belgium), eBioscience (ebioscience, Schwerte, Germany), Biolegend (Biolegend, London, United Kingdom), Invitrogen (Invitrogen, Schwerte, Germany), and Beckman Coulter (Beckman Coulter, Krefeld, Germany). Following dyes were used for the Treg surface phenotyping: anti-CD4 (mouse IgG1, PerCP), anti-CD8 (mouse IgG1, APC-H7), anti-CD25 (mouse IgG1, PE-Cy7), anti-CD127 (mouse IgG1, FITC), and appropriate isotype controls. Cytokine assessment included: anti-CD3 (mouse IgG1, HorV450), anti-CD8 (mouse IgG1, APC-H7), anti-IL-2 (mouse IgG1, PE), anti-IL-10 (rat IgG1, APC), anti-IL-17A (mouse IgG1, PerCP), anti-IFNγ (mouse IgG1, FITC), anti-TNFα (mouse IgG1, PE), and anti-CD69 (mouse IgG1, PE-Cy7). We carried out flow cytometry acquisition on a 3-laser Navios instrument (Beckman Coulter), equipped to detect 10 fluorescent parameters. Compensation and data analyses were done with Kaluza Analysis 1.5a software (Beckman Coulter).

### T-Cell Quantification

Absolute counts of CD3^+^ T-cells, CD4^+^ T-cells, and CD8^+^ T-cells were assessed in lysed whole blood immediately after collection. 100 µl of an undiluted blood sample was stained with premixed multitest antibody anti-CD3 (mouse IgG1, PE-PC7), anti-CD4 (mouse IgG1, PE), and anti-CD8 (mouse IgG1, FITC) (Beckman Coulter), vortexed and lysed (Beckman Coulter). 100 µl AccuCheck Counting Beads (Thermo Fisher scientific, Schwerte, Germany) were mixed with the sample to allow absolute quantification by flow cytometry.

### T-Cell Analysis

Surface phenotyping and intracellular cytokine analyses of T-cells were performed with freshly isolated PBMC. PBMCs were stained with antibodies for 30 min and analyzed immediately after washing with Dulbecco’s phosphate-buffered saline (DPBS 1×; Gibco, Life Technologies) in case of surface phenotyping. Isotype controls were used to confirm specificity of staining and to discriminate background staining.

To assess cytokine production of T-cells, PBMCs were stimulated with a stimulation cocktail (plus protein transport inhibitors; Ebioscience) for 4 h. Stimulation cocktail consists of phorbol-12-myristate 13-acetate (PMA), ionomycin, brefeldin A, and monensin as protein transport inhibitors. Surface staining, fixation, and permeabilization (CytoFix/CytoPerm kit; BD Biosciences) followed. After fixation and permeabilization, PBMCs were stained intracellularly for IFNγ, IL-2, IL-10, IL-17A, and/or TNFα. Unstimulated PBMC served as controls. Stimulation induces a downregulation of CD4, thus CD4^+^ T-helper-cells (THs) were defined as CD3^+^CD8^neg^ T-cells.

### Peripheral Cytokine Level

EDTA plasma concentrations of IL-10, TNFα, and IP-10 were measured by ELISA (Human Quantikine ELISA, R&D Systems, Wiesbaden-Nordenstadt, Germany) at room temperature on a Fluostar Optima microplate reader (BMG Labtech, Offenburg, Germany). The sensitivity of the assays was 3.9 pg/ml for IL-10, 0.7 pg/ml for TNFα, and 4.46 pg/ml for IP-10.

### Statistical Analysis

Mean values, their ±SEM and ranges, and normal distribution of data were calculated for each variable using the SPSS Software (SPSS 22.0, SPSS Inc., Chicago, IL, USA). Grubbs’ test was used to identify outliers. The graphs were made using GraphPad Prism^®^ 6 (Version 6.01, GraphPad Software, Inc., La Jolla, CA, USA). Repeated measures (e.g., cytokine concentrations) were compared between LPS and placebo conditions using two-way repeated measure ANOVA with the repeated factors time and condition (i.e., LPS, placebo). To compare single measurement points separately between LPS and placebo, *post hoc* paired *t*-tests were calculated with Bonferroni corrections for multiple testing, if not otherwise indicated. The *p*-values <0.05 were considered to be statistically significant.

## Results

### Demographic and Clinical Characteristics

Twenty healthy volunteers of Caucasian ethnicity with a mean age of 26.1 ± 0.9 years (range: 18–34) and a mean BMI of 24.2 ± 0.5 kg/m^2^ (range: 19.3–26.9) were included in this cross-over study. There was no difference with respect to the time to switch conditions comparing the two groups (LPS/placebo vs. placebo/LPS, 32 ± 11.4 days vs. 33 ± 8.4 days, *p* = 0.86). We did not observe differences between participants who received LPS before or after saline in any dependent variable (data not shown). Endotoxin injection induced a transient systemic inflammatory response in all participants, which was characterized by a significant increase in WBC count. In addition, body temperature, heart rate, and respiratory rate increased after LPS administration (all *p* < 0.001, ANOVA interaction effects, Table 1).

**Table 1:** Clinical parameters at baseline and 3 h after LPS/placebo injection.

Variables	LPS condition (*n* = 20)	Placebo condition (*n* = 20)	Interaction *p*-value
Body temperature (°C)	36.2 (±0.1) vs. 37.6 (±0.1)**	36.3 (±0.1) vs. 36.4 (±0.1)	<*0.001*
BP systolic (mmHg)	130.0 (±2.6) vs. 116.4 (±6.0)	131.2 (±3.0) vs.127.3 (±2.6)	0.248
BP diastolic (mmHg)	68.1 (±1.6) vs. 63.1 (±1.9)	70.5 (±1.8) vs. 66.0 (±1.8)	0.844
HR (bpm)	72.3 (±2.5) vs. 85.2 (±3.2)**	73.2 (±2.9) vs.68.5 (±2.3)	<*0.001*
Respiratory rate (ipm)	14.3 (±0.4) vs.16.0 (±0.5)**	14.8 (±0.4) vs.14.4 (±0.4)	<*0.001*
WBC count (×10^3^/μl)	5.3 (±0.3) vs.8.3 (±0.5)**	5.1 (±0.2) vs. 5.7 (±0.2)**	<*0.001*
HCT (%)	39.1 (±0.5) vs. 38.8 (±0.7)	39.9 (±0.9) vs. 38.4 (±0.8)*	0.120

*Data were presented as mean ± SEM. Data were analyzed with two-way ANOVA, followed by exploratory paired t-tests*.

*LPS, lipopolysaccharide; HR, heart rate; BP, blood pressure; WBC, white blood cell; HCT, hematocrit. Interaction p-values in italics are significant*.

Asterisks indicate the significance level of the paired t-test: *<0.05, **<0.005, no asterisk, not significant.

### Cellular Response to LPS Injection

**Figure 1:**
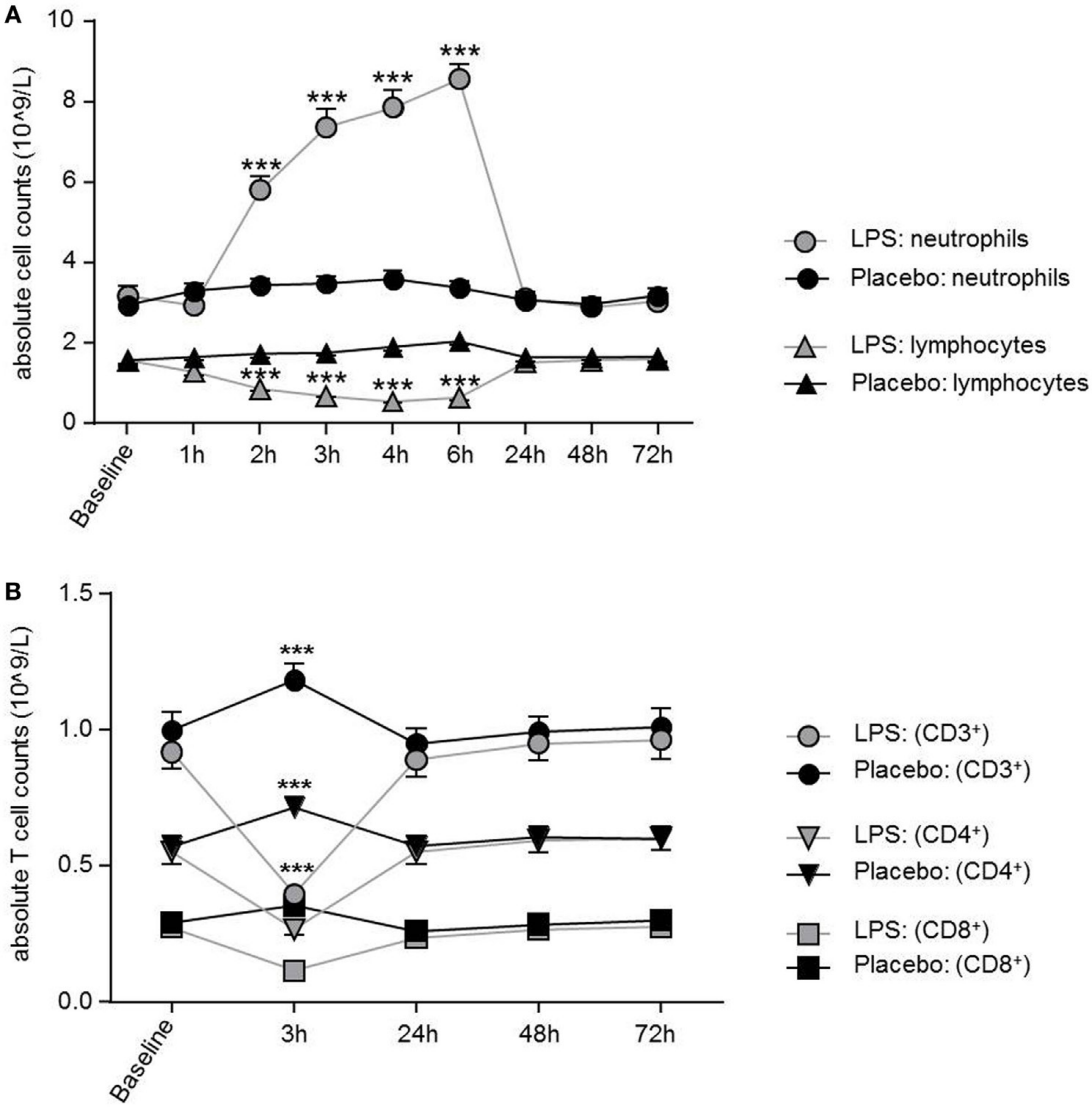
Dynamics of neutrophils, lymphocytes, and T-cells over time after lipopolysaccharide (LPS) challenge. **(A)** Neutrophils were significantly increased between 2 and 6 h after LPS application and normalized after 24 h, while lymphocytes showed a significant decrease between 2 and 6 h. Neutrophil and lymphocyte counts remained unchanged in the placebo condition. **(B)** After 3 h of LPS injection, all T-cell subsets showed a significant decrease of absolute T-cells counts with normalization after 24 h. In the placebo condition, T-cell subset counts were increased 3 h after placebo injection as compared to baseline, following the circadian rhythm (nadir of naïve CD4^+^T-cells/naïve CD8^+^ cytotoxic T-cells at 11:00 a.m., peak of naïve CD4^+^ T-cells/CD8^+^ T-cells around 2:00 a.m.) Data are given as mean (±SEM). Two-way ANOVA analysis with repeated measures was performed followed by *post hoc* Bonferroni-corrected paired *t*-tests. *p < 0.05, **p < 0.01, ***p < 0.001, results of *post hoc* Bonferroni-corrected *t*-test. For results of ANOVA see text.

LPS application led to a significant increase in total neutrophil count peaking at 6 h post-injection (*F* = 92.51, *p* < 0.001; ANOVA interaction effect, Figure 1A), while monocytes (*F* = 14.16; *p* < 0.001; ANOVA interaction effect, data not shown) and lymphocytes (*F* = 108.8; *p* < 0.001; Figure 1A) significantly decreased. Maximum reduction of lymphocyte and monocyte counts occurred 4 or 2 h after LPS injection. Cell counts were normalized after 24 h for lymphocytes and after 4 h for monocytes. The absolute numbers of circulating T-cells were diminished 3 h after LPS challenge and recovered at 24 h (Figure 1B).

### Soluble Systemic IL-10 and IP-10 Levels Increase After LPS Challenge

**Figure 2:**
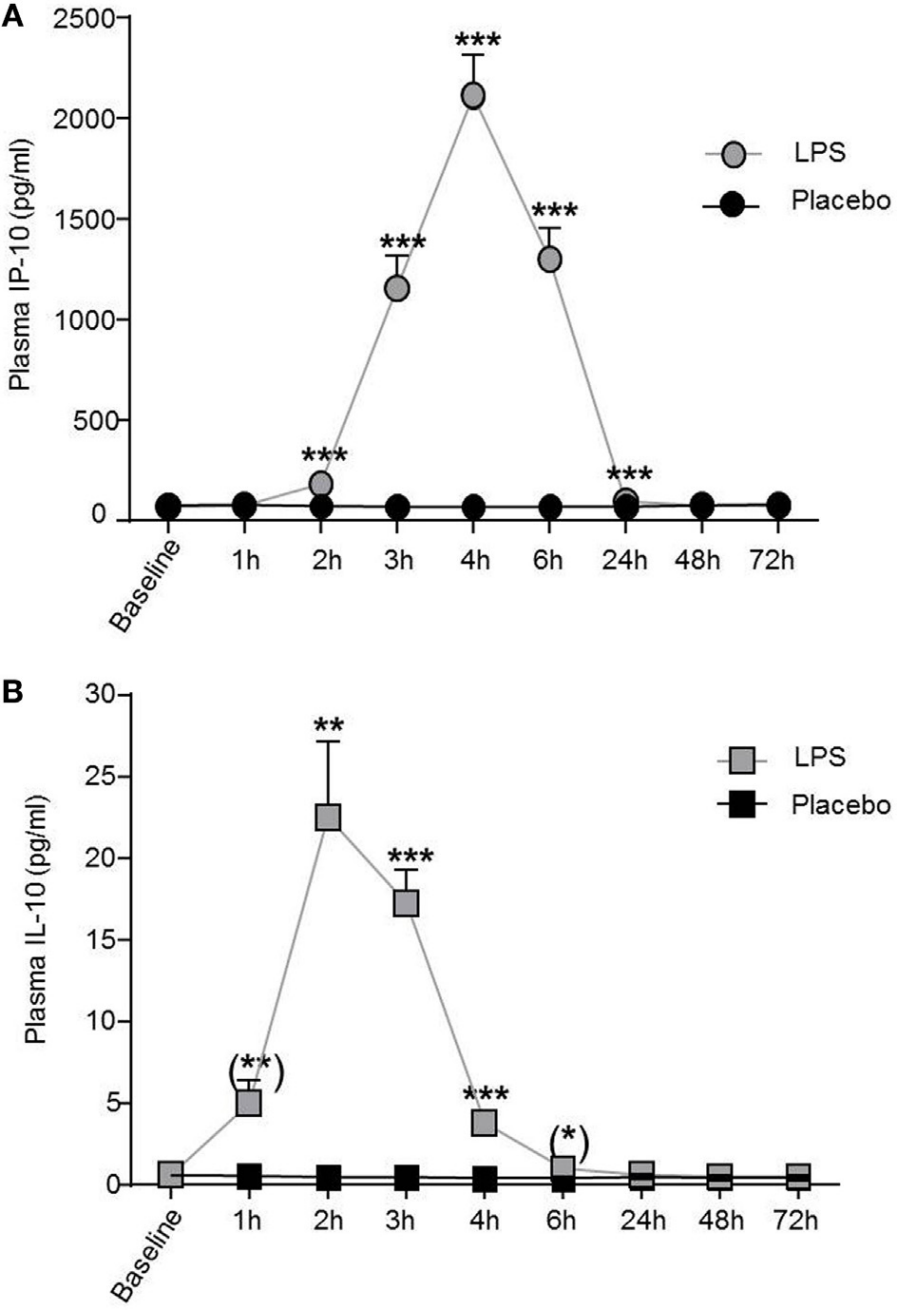
IP-10 and IL-10 plasma concentrations increased after lipopolysaccharide (LPS) challenge. **(A)** Pro-inflammatory cytokine plasma levels of IP-10 and **(B)** of the anti-inflammatory cytokine IL-10 increased after LPS (0.8 ng/kg, gray boxes) challenge. Placebo administration (black boxes) did not alter IP-10 or IL-10 plasma levels. Mean ± SEM are shown. Two-way ANOVA analysis was performed with repeated measures followed by *post hoc* Bonferroni-corrected paired *t*-tests. *p < 0.05, **p < 0.01, ***p < 0.001, results of *post hoc* Bonferroni-corrected paired *t*-tests. For results of ANOVA, see text. Asterisks in parentheses indicate results from *post hoc* paired *t*-test, which remain non-significant after Bonferroni correction.

Low-dose LPS injection induced significant changes in plasma concentrations of the pro-inflammatory cytokines interferon-induced protein 10 and TNFα (IP-10, *F* = 72.0, *p* < 0.001, Figure 2A; TNFα, *F* = 26.2, *p* < 0.001, both ANOVA interaction effects), as well as in the anti-inflammatory cytokine IL-10 (*F* = 21.5, *p* < 0.001; Figure 2B). The maximum IP-10 concentration was reached 4 h after LPS administration, whereas the IL-10 peak concentration occurred earlier at 2 h after LPS injection. IP-10 and IL-10 levels returned to baseline after 24 h (Figure 2).

### Effector T-Cells Fail to Produce Pro-Inflammatory Cytokines After LPS Challenge Whereas Anti-Inflammatory Treg Remain Unaffected

**Figure 3:**
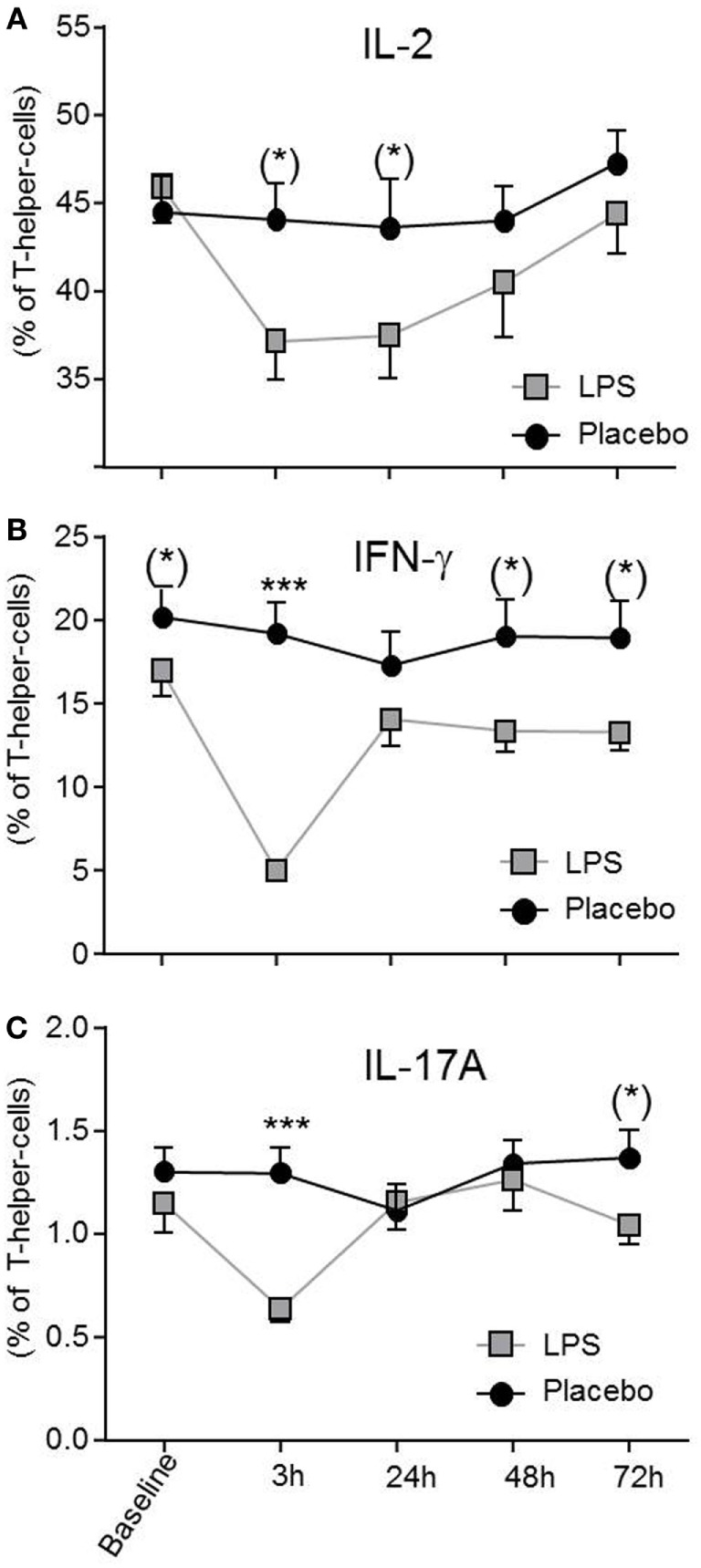
Circulating effector T-helper-cells (THs) are diminished after lipopolysaccharide (LPS) challenge. THs producing IL-2 **(A)**, IFNγ **(B)**, and IL-17A **(C)** after LPS- (gray boxes) or placebo administration (black circles) are given as mean ± SEM over 72 h. IFNγ and IL-17A producing THs were significantly decreased 3 h after LPS injection and returned to baseline after 24 h. Two-way ANOVA were performed with repeated measures followed by *post hoc* Bonferroni-corrected paired *t*-tests. *p < 0.05, **p < 0.01, ***p < 0.001, results of *post hoc* Bonferroni-corrected paired *t*-tests. For interaction *p*-values of ANOVA, see text. Asterisks in parentheses indicate results from *post hoc* paired *t*-test, which remain non-significant after Bonferroni-correction.

**Figure 4:**
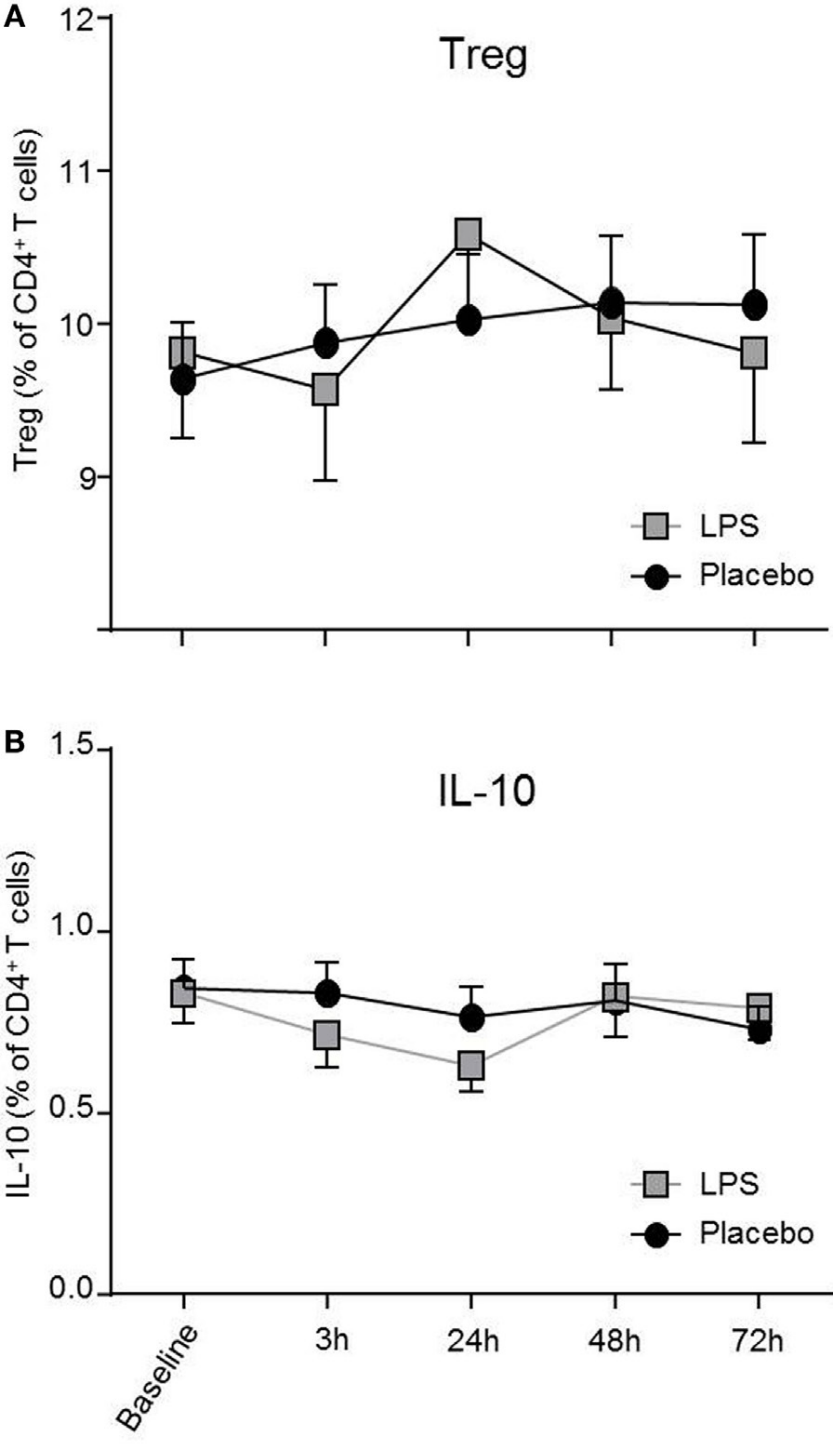
Treg numbers and IL-10 production by T-helper-cells (THs) are not impaired after lipopolysaccharide (LPS) challenge. CD4^+^CD25^hi^CD127^low^ Treg **(A)** and IL-10 producing THs **(B)** after LPS-(gray boxes) or placebo administration (black circles) are depicted as mean percentages (±SEM) over 72 h. Two-way ANOVA with repeated measures were performed. The *p*-value compares the interaction between time and condition.

Total number of CD3^+^ T-cells (*F* = 42.4, *p* < 0.001; Figure 1B), CD4^+^ T-cells (*F* = 40.4, *p* < 0.001; Figure 1B), and CD8^+^ T-cells (*F* = 22.3, *p* < 0.001, all ANOVA interaction effects; Figure 1B) showed a significant reduction 3 h after LPS injection and normalized after 24 h. In the placebo condition, changes in T-cell subset counts were observable according to the circadian rhythm (21). Interestingly, the percentage of IL-2 (*F* = 2.2, *p* = 0.08, Figure 3), IFNγ (*F* = 3.7, *p* < 0.001, Figure 3), TNFα (*F* = 3.87, *p* = 0.007, data not shown), and IL-17A (*F* = 7.4, *p* < 0.008, Figure 3; Table 2) producing THs decreased 3 h after LPS injection as compared to baseline and placebo (all ANOVA interaction effects). IFNγ and IL-17A production by THs were fully restored 24 h after LPS injection (Figure 3). In contrast, the relative fraction of Treg was stable and remained unchanged after LPS injection (*F* = 1.8, *p* = 0.13; Figure 4). The absolute numbers of Treg were reduced due to the drop of total T-cell numbers after LPS challenge. Nevertheless, the capacity of Treg to produce IL-10 was not affected by LPS challenge (*F* = 0.7, *p* = 0.57; ANOVA interaction effects, Figure 4).

**Table 2:** Immunological characterization: T-cell cytokine production after LPS/placebo injection.

Intracellular cytokine production	LPS condition (*n* = 20)	Placebo condition (*n* = 20)	Interaction *p*-value
	0 vs. +3 h	0 vs. +3 h	
	0 vs. +24 h	0 vs. +24 h	
IL-2 (% of THs)	45.4 (±2.0) vs. 37.2 (±2.2)**	43.9 (±2.1) vs. 44.1 (±2.1)	*0.008*
	46.0 (±2.1) vs. 37.8 (±2.4)**	44.5 (±2.1) vs. 43.6 (±2.8)	*0.023*
IL-10 (% of THs)	0.84 (±0.1) vs. 0.71 (±0.1)	0.84 (±0.1) vs. 0.83 (±0.1)	0.399
	0.83 (±0.1) vs.0.64 (±0.1)	0.84 (±0.1) vs. 0.76 (±0.1)	0.249
IL-17A (% of THs)	1.15 (±0.1) vs. 0.63 (±0.1)**	1.28 (±0.1) vs. 1.30 (±0.1)	*0.002*
	1.15 (±0.1) vs. 1.18 (±0.1)	1.30 (±0.1) vs. 1.11 (±0.1)	*0*.075
IFNγ (% of THs)	17.3 (±1.5) vs. 5.0 (±0.7)**	19.9 (±1.7) vs.19.3 (±1.8)	<*0.001*
	17.3 (±1.5) vs. 14.2 (±1.6)	20.3 (±1.7) vs. 17.2 (±2.0)	0.961

*Data were presented as mean ± SEM. The percentage of THs producing IL-2, IL-10, IL-17A, or IFNγ is given. Data were analyzed with two-way ANOVA, followed by exploratory paired t-tests*.

Asterisks indicate the significance level of the paired t-test: *<0.05, **<0.005, no asterisk, not significant. Interaction p-values in italics are significant.

*LPS, lipopolysaccharide; IL, interleukin; THs, T-helper-cells*.

## Discussion

This study reveals new insights into the differential effects of endotoxemia on anti- and pro-inflammatory T-cell responses. Overall, absolute T-cell numbers were decreased sharply after LPS challenge within 3 h after injection. Pro-inflammatory effector THs lost the capacity to produce IL-17A, IL-2, TNFα, and IFNγ as early as 3 h after LPS injection and regained function post endotoxemia. The relative number of anti-inflammatory Tregs and its capacity to produce IL-10 remained stable and did not change during endotoxemia.

In contrast to murine experimental endotoxemia, humans are highly sensitive to LPS (2, 4). Thus, healthy participants challenged with LPS respond with a rise of body temperature and fever, elevated heart, and breathing rate partially leading to tachycardia/tachypnea, decreased blood pressure, and neutrophilia (11, 22–25). Human experimental endotoxemia is a well-investigated model for systemic inflammation (7–13, 26, 27). This model is advantageous over patient studies, as it is not biased by pretreatment or comorbidities. The experimental human endotoxemia model has been used in low and high dose settings to address different immunological questions regarding innate and cellular immune responses during systemic inflammation (0.4–4.0 ng/kg bodyweight) (7, 11, 14, 22, 24–27).

In our cohort, LPS challenge in the lower dosage range caused a clinical and an immunological response of the subjects, which was absent under placebo conditions indicating the efficacy of our model.

There was a differential effect on T-cell populations in our model. The capacity of effector T-cells to produce pro-inflammatory cytokines such as IFNγ, TNFα, IL-2, or IL-17A was diminished 3 h after LPS injection. Impaired IFNγ production by T-cells has been described after administration of higher doses of LPS (2.0 and 4.0 ng/kg bodyweight); in line with our study, IFNγ production normalized after 24 h (13, 22). Whereas in these studies, cytokine concentrations in supernatants were determined, we clearly demonstrated a lack of function and impairment of the circulating CD4^+^ TH compartment to produce IFNγ on single-cell level (13, 22). Van de Veerdonk et al. reported deficient *Candida*-specific IL-17A^+^ T-cell responses in healthy subjects receiving a relatively high dose of LPS (2 ng/kg bodyweight) and in patients with gram negative sepsis (28). We found that even a lower dose of LPS led to a reduced capacity of effector T-cells to produce IL-17A; however, this effect was transient and effector T-cells recovered 24 h after LPS challenge.

We further studied potential causes for the dysfunction of the effector TH compartment. Increased numbers of or enhanced activity of anti-inflammatory Treg may lead to potent inhibition of effector T-cells (18, 19). LPS exposure may have a direct effect on Treg and is reported to promote Treg function (29, 30). Interestingly, neither the relative fraction nor the functional capacity of Treg to produce IL-10 was altered after LPS administration in our study. Ronit et al. observed a relative increase of Treg, which returned to baseline 24 h after LPS injection (14). In addition, the authors reported a marked suppression of a broad range of cytokines, including IL-10, when whole blood was stimulated with the T-cell mitogen phytohemagglutinin. These results conflict with our findings. We found IL-10 production to be unaffected on single T-cell level and demonstrated that the relative fraction of Treg remained stable after LPS challenge. However, there are several methodological differences between the study of Ronit et al. and ours, which may explain the observed discrepancies. First, the dosage of LPS administered to healthy volunteers was much lower in our study. Second, we chose, in contrast to Ronit et al., a placebo-controlled cross-over study design and followed a higher number of healthy volunteers in a longitudinal manner. Finally, we determined and characterized T-cells on a functional single-cell level, whereas Ronit et al. did not determine the functional capacity of T-cells but instead measured cytokines in supernatants of stimulated whole blood cultures (14). Nevertheless, in our cohort systemic levels of the anti-inflammatory cytokine, IL-10 increased significantly during endotoxemia and might be derived from other cells than T-cells. Accordingly, LPS also promotes IL-10 secretion by neutrophils (29, 30). Thus, impaired capacity of T-cells to exert pro-inflammatory function might be caused by soluble factors such as IL-10 released by LPS-triggered neutrophils or monocytes. In line with this observation, T-cell capacity to produce IFNγ or IL-17A was normalized after 24 h when systemic IL-10 levels had returned to their baseline. It has to be considered that other cell subsets not being investigated in our study, such as unconventional T-cell subsets, may mediate additional anti-inflammatory effects (31, 32).

Alternatively, it has to be considered that pro-inflammatory T-cells were not rendered anergic but were simply depleted from the circulation by increased apoptosis, pooling in the spleen, or increased adhesion to vessel walls (4, 33, 34). In addition, T-cells may migrate to peripheral tissues such as lung or liver during systemic inflammation. Depletion or migration of T-cells would have prevented detection of pro-inflammatory T-cell subsets in our *ex vivo* assay.

The limitation of our study is that a single endotoxin challenge was administered; thus, we investigated in our model the immune response to an acute challenge of LPS which does not resemble sepsis. Accordingly, immunosuppression in our experimental human endotoxemia model was transient and short-lived in contrast to persistent, long-lasting immunoparalysis in sepsis (4, 7, 11, 14). Kox et al. studied earlier the effect of repeated LPS challenges in healthy volunteers (35). A diminished *in vivo* response to LPS in terms of attenuated TNFα-, IL-6, and IL-10 serum levels was observed upon re-challenge. The *ex vivo* T-cell response has not been assessed in these kinds of studies, and it will be of interest to know whether repeated LPS challenges have similar impact on T-cell function. Furthermore, immunoparalysis is usually observed days after onset of sepsis whereas transient immunosuppression in the human endotoxemia model is already detectable after hours. Therefore, the immunological response to a single endotoxin challenge in the human experimental endotoxemia model reflects a physiological mechanism to limit the immune response after an initial pro-inflammatory phase rather than a dysregulation of the immune system. Notwithstanding, the mechanisms inducing the immunosuppressive state are assumed to be similar and thus, this model is also used in interventional trials to assess pharmacological interventions to abrogate immunoparalysis (7). Our findings that Th1 and Th17 cells are profoundly suppressed by endotoxemia whereas IL-10 production by T-cells remains unaffected, may add to the understanding dysregulated immunity sepsis. Indeed, a post-mortem study revealed that production of Th1-associated cytokines by splenic T-cells from septic patients was significantly reduced (6). In addition, diminished Th17 cell responses have been reported in patients with sepsis (28). Another study assessed T-cell-derived IL-10 production and found no difference between septic patients and controls, which is in line with our data (36). Altogether, this may suggest that restoration of pro-inflammatory immunity is more important than abrogation of anti-inflammatory immunity as future therapeutic strategy to break immunoparalysis.

In conclusion, we could demonstrate that even a low dose of LPS induces potent suppression of pro-inflammatory T-cell subsets and does not affect the capacity of anti-inflammatory Treg to produce IL-10.

## Ethics Statement

This study was carried out in accordance with the recommendations of the local ethics committee of the University Hospital Essen, Germany. The protocol was approved by the local ethics committee of the University Hospital Essen, Germany (15-6533-BO). All subjects gave written informed consent in accordance with the Declaration of Helsinki.

## Author Contributions

AB designed the study, performed the experiments, performed data analysis, and wrote the manuscript. AS and JK performed the experiments, performed data analysis, and wrote the manuscript. HE, SD, and SB performed data analysis and wrote the manuscript. MS and AK designed the study and wrote the manuscript. OW and BW designed the study, performed data analysis, and wrote the manuscript.

## Conflict of Interest Statement

The authors declare that the research was conducted in the absence of any commercial or financial relationships that could be construed as a potential conflict of interest.
